# Early Inpatient Rehabilitation Following Neuromyelitis Optica Spectrum Disorder Flare: A Case Report

**DOI:** 10.7759/cureus.113625

**Published:** 2026-07-29

**Authors:** Darren H Tom, Tha Cha

**Affiliations:** 1 Physical Medicine and Rehabilitation, California Health Sciences University College of Osteopathic Medicine, Clovis, USA; 2 Physical Medicine and Rehabilitation, San Joaquin Valley Rehabilitation Hospital, Fresno, USA

**Keywords:** demyelination, inpatient rehabilitation, neuromyelitis optica, neuroplasticity, nmosd, rehabilitation, remyelination, stroke recovery

## Abstract

Neuromyelitis optica spectrum disorder (NMOSD) is a rare autoimmune demyelinating disease primarily affecting the central nervous system. Relapsing NMOSD flares can result in severe functional decline, including paraplegia and vision loss. We present a 46-year-old woman admitted to an acute inpatient rehabilitation facility (IRF) following a clinically suspected NMOSD flare triggered by aspiration pneumonia. During a two-week admission, the patient demonstrated significant improvement in activities of daily living (ADLs) and Section GG functional scores with a multidisciplinary rehabilitation program consisting of approximately three hours of therapy per day over five days. This case highlights the potential role of early, intensive inpatient rehabilitation following a clinically suspected NMOSD flare in optimizing neurologic recovery and functional outcomes. It may also provide a framework for applying stroke-based neurorehabilitation models that emphasize early intervention and activity-dependent neuroplasticity during neurologic recovery in patients with NMOSD.

## Introduction

Neuromyelitis optica spectrum disorder (NMOSD) is a rare, relapsing autoimmune demyelinating condition predominantly affecting the optic nerves and spinal cord. It is distinguished from multiple sclerosis via its association with aquaporin-4 IgG (AQP4-IgG) antibodies, which are highly specific for NMOSD [1,2]. Pathophysiologically, AQP4-IgG binding to astrocytes triggers complement-mediated injury, leading to secondary demyelination and neuronal dysfunction [3]. The resulting disruption of motor, sensory, and coordination pathways can produce abrupt focal neurologic deficits similar to those seen following other acute central nervous system injuries, including stroke.

Relapsing flares in NMOSD often lead to respiratory failure, mortality, and abrupt neurologic deficits such as vision loss, paraplegia, bladder dysfunction, neuropathic pain, altered level of consciousness, and long-term disability [1,4]. Additionally, up to 30% of flare events may be preceded or triggered by systemic stressors such as infection [1]. These flares of NMOSD produce focal neurologic deficits that translate into substantial impairments in mobility and activities of daily living (ADLs).

Immunologic therapies such as high-dose steroids, plasmapheresis, and maintenance immunosuppressants remain the cornerstone of relapse management [4]. However, these interventions do not directly address restoration of strength, balance, or functional independence. In contrast, neurologic rehabilitation research, especially in the context of stroke, emphasizes that the first 8-12 weeks following an acute neurologic insult represent a critical window during which neuroplastic reorganization is most active [5,6]. During this period, heightened synaptic plasticity and cortical reorganization are thought to increase responsiveness to task-specific rehabilitation, thereby maximizing functional gains.

The neuroplasticity literature further supports the concept that early, task-specific rehabilitation promotes adaptive cortical reorganization during the recovery window following acute neurologic injury [7]. Finally, early, intense, task-specific therapy in inpatient settings has repeatedly been shown to outperform delayed or lower-intensity models in stroke populations [8].

While NMOSD is primarily an autoimmune astrocytopathy, and stroke is an ischemic injury, both conditions ultimately disrupt descending motor pathways and white matter networks responsible for motor learning and functional movement [1,9]. Emerging evidence suggests that ischemic stroke can result in secondary oligodendrocyte injury, demyelination, and impaired white matter integrity, with remyelination contributing to neurologic recovery [10]. These shared downstream mechanisms provide a biologically plausible rationale for extrapolating certain neuroplasticity-based rehabilitation principles from stroke to NMOSD, although direct evidence supporting equivalent recovery mechanisms remains limited. Thus, rehabilitation strategies that leverage activity-dependent neuroplasticity and repetitive task-specific training may reasonably apply to NMOSD recovery following acute relapse. This case report highlights an early inpatient rehabilitation approach following a clinically suspected NMOSD flare, with functional recovery tracked using Section GG functional scores.

## Case presentation

A 46-year-old woman with a 15-year history of NMOSD was admitted to an inpatient rehabilitation facility (IRF) in mid-April for functional decline following a clinically suspected NMOSD flare. Following aspiration pneumonia in late March, the patient developed progressive neurologic symptoms consistent with a clinically suspected NMOSD flare, including left-predominant weakness, worsening gait instability with left knee buckling, and decreased vision in the left eye. She underwent acute hospitalization followed by a brief stay at a skilled nursing facility (SNF) with minimal functional improvement before being transferred to the inpatient rehabilitation facility for intensive rehabilitation approximately three weeks after symptom onset in late March.

Her prior level of function included independence with ADLs, transfers, and ambulation without assistive devices. Historically, she recovered within five days after mild flares when treated with corticosteroids. During her seven-day hospitalization, she was treated with corticosteroids and trimethoprim-sulfamethoxazole before being discharged to a skilled nursing facility (SNF) for about 14 days. However, this episode was prolonged and did not result in significant functional improvement during the SNF stay.

The relapse was clinically suspected rather than radiographically confirmed, based on the patient's established history of NMOSD, acute neurologic deterioration in the setting of aspiration pneumonia, and symptom patterns consistent with prior flares. The combination of asymmetric bilateral weakness, greater on the left than the right, and decreased vision in the left eye was consistent with NMOSD, more specifically, optic neuritis, which can cause decreased visual acuity, and longitudinally extensive transverse myelitis (LETM), which can produce asymmetric bilateral motor deficits due to the involvement of multiple contiguous spinal cord segments. Advanced imaging and serologic testing were not obtained before or during inpatient rehabilitation due to resource limitations.

The referring SNF provided documents before the patient's transfer to the inpatient rehabilitation facility, describing gait impairment and ambulation of approximately 75 feet with moderate assistance. However, upon admission to the IRF, the patient was able to walk only 28 feet with a front-wheeled walker and moderate assistance. Additionally, she needed to sit down in the wheelchair following her once for safety due to leg spasms. Neurologic examination revealed left-predominant bilateral weakness (left lower extremity 3-4/5, left upper extremity 4/5, right-sided strength of both upper and lower extremities 4/5), left knee buckling during ambulation, bilateral shoulder spasticity, and persistent decreased vision in the left eye.

Initial admission Section GG self-care scores (Table 1) demonstrated setup assistance (GG 05) for eating, oral hygiene, and upper extremity dressing. In contrast, the patient remained dependent (GG 01) for toileting hygiene, bathing, lower extremity dressing, and footwear management. Section GG mobility scores (Table 2) showed dependence (GG 01) in negotiating one-step curbs, toilet transfers, and shower transfers. Chair transfers, bed mobility (including rolling, sit-to-lie, and lie-to-sit), sit-to-stand, walking 10 feet, and wheelchair propulsion over 50 feet with two turns required partial/moderate assistance (GG 03). Car transfers, object retrieval, and other mobility tasks, such as negotiating a four-step curb, walking on uneven surfaces, and walking more than 50 feet, were deferred due to either patient safety or medical condition (GG 88).

**Table 1:** Section GG self-care scores at admission and discharge 01 = Dependent: Helper provides all effort or assistance of two or more helpers is required. 02 = Substantial/maximal assistance: Patient performs less than 50% of the activity. 03 = Partial/moderate assistance: Patient performs more than 50% of the activity. 04 = Supervision or touching assistance: Verbal cues, supervision, or contact guard assistance only. 05 = Setup or clean-up assistance: Helper prepares or cleans up; patient completes task independently. 06 = Independent: Patient completes the activity without assistance.

Functional domain	Admission score	Discharge score	Change
Eating	5	6	1
Oral hygiene	5	6	1
Toileting hygiene	1	2	1
Shower/bathe self	1	3	2
Upper extremity dressing	5	5	0
Lower extremity dressing	1	3	2
Putting on/taking off footwear	1	3	2

**Table 2:** Section GG mobility scores at admission and discharge 88 = Not attempted due to medical condition or safety concern. 01 = Dependent: Helper provides all effort or assistance of two or more helpers is required. 02 = Substantial/maximal assistance: Patient performs less than 50% of the activity. 03 = Partial/moderate assistance: Patient performs more than 50% of the activity. 04 = Supervision or touching assistance: Verbal cues, supervision, or contact guard assistance only. 05 = Setup or clean-up assistance: Helper prepares or cleans up; patient completes task independently. 06 = Independent: Patient completes the activity without assistance.

Functional domain	Admission score	Discharge score	Change
Rolling	3	6	3
Sit to lying	3	6	3
Lying to sitting	3	6	3
Sit to stand	3	4	1
Toilet transfer	1	4	3
Shower transfer	1	4	3
Chair transfer	3	4	1
Car transfer	88	4	4
Pick up objects	88	4	4
Walk 10 feet	3	4	1
Walk 10 feet - uneven surface	88	88	0
Walk 50 ft with two turns	88	88	0
Walk 150 feet	88	88	0
1 step curb	1	4	3
4 step curb	88	88	0
Wheel 50 feet with two turns	3	4	1
Wheel 150 feet	88	88	0

On initial physical therapy (PT) evaluation, the patient demonstrated fair-minus trunk control and poor-plus dynamic balance, indicating the ability to maintain sitting balance with minimal assistance and initiate weight shifting with moderate assistance, but without the capacity to sustain stability independently during dynamic tasks.

She was initially treated with an oral corticosteroid taper and baclofen for spasticity. She also participated in approximately 3 hours of combined rehabilitation 5 days per week, consisting of 120 minutes of PT, 60 minutes of occupational therapy (OT), and daily rehabilitation nursing focused on bowel/bladder management, medication education, skin protection, and reinforcement of transfer and mobility strategies. Therapy interventions focused on gait and transfer training, balance and postural control activities, strengthening exercises, ADL retraining, and stretching to address spasticity and functional mobility limitations.

Halfway through her admission, the patient began to experience more frequent spasms in her lower extremities. Baclofen dosing was increased, but spasticity did not improve. Thus, the decision was made to discontinue baclofen and start cyclobenzaprine, which led to initial improvement in spasticity. However, the patient continued to have spasticity, and the cyclobenzaprine dosage was further increased. The increased dosage was sufficient to provide relief during spasticity episodes and continue scheduled therapy.

Around day 10 of admission, the patient developed worsening neck heaviness and fatigue that were felt by the multidisciplinary team to be consistent with a clinically suspected second flare based on her history of NMOSD and similarity to prior relapse symptoms. No new focal neurologic deficits were noted, and confirmatory neuroimaging, cerebrospinal fluid studies, or serologic testing were not obtained due to resource limitations. Consequently, the episode was managed as a clinically suspected relapse rather than a radiographically confirmed flare with high-dose corticosteroids.

During episodes of spasticity and the second flare, therapy was modified to include more stretching activities and to shorten or skip sessions, as needed, based on the patient's tolerance. The patient was able to complete most sessions, except for one day when they missed the second PT session. However, the patient was able to complete an OT session and the first PT session, which included more stretching and mobility exercises.

At discharge on day 14, the neurologic examination revealed improved strength (left lower extremity 4/5, left upper extremity 4-/5, right upper and lower extremities 4+/5), minimal left knee buckling during ambulation, transient bilateral shoulder spasticity controlled with cyclobenzaprine, and transient decreased vision in the left eye. The patient displayed improved ambulation, walking 35 feet with a front-wheel walker and touch assistance.

Section GG scores demonstrated meaningful functional improvement across multiple self-care (Table 1) and mobility (Table 2) domains when compared to admission. The patient achieved independence (GG 06) in eating, oral hygiene, and bed mobility (rolling, sit-to-lie, and lie-to-sit). Transfers, including sit-to-stand, car transfer, chair transfer, toilet transfer, and shower transfer, improved to supervision or touching assistance (GG 04), as did walking 10 feet, negotiating a one-step curb, wheelchair propulsion for 50 feet with two turns, and object retrieval. Lower extremity dressing, bathing, and footwear management improved from dependent status (GG 01) at admission to partial/moderate assistance (GG 03), while toileting hygiene improved to substantial/maximal assistance (GG 02). Mobility activities, including negotiating a four-step curb, walking on uneven surfaces, and walking more than 50 feet, were again deferred due to either patient safety concerns or the medical condition (GG 88).

PT evaluations also demonstrated improved trunk control, balance, and postural stability, with fair performance during both static and dynamic sitting and standing activities.

Overall, intensive inpatient rehabilitation began approximately three weeks after symptom onset and continued for 14 days, placing the patient's rehabilitation course well within the proposed first 8-12-week neuroplastic recovery window discussed in the Introduction.

She was then discharged home after final evaluation with extensive family support, durable medical equipment (DME), medication management, home PT, and follow-up arrangements with her primary care provider.

## Discussion

NMOSD presents a unique challenge for rehabilitation, as recurrent inflammatory demyelination often leads to cumulative motor, sensory, and visual deficits. However, recovery potential in NMOSD may be greater than previously assumed, secondary to the use of neuroplastic mechanisms similar to those observed in stroke rehabilitation [7]. The first 8-12 weeks following a neurologic insult, whether ischemic, traumatic, or demyelinating, represent a critical window for motor relearning and synaptic reorganization [5,6,11]. Evidence from stroke recovery research suggests that this period is the most responsive phase for promoting neuroplasticity through task-specific and repetitive training [7,12,13].

Although NMOSD and ischemic stroke differ in their underlying etiologies, both ultimately disrupt white matter tracts responsible for motor function. Experimental and translational studies have shown that ischemic stroke produces secondary oligodendrocyte injury, demyelination, and impaired white matter integrity, with remyelination recognized as an important component of neurologic recovery [9]. Furthermore, microglia-mediated regulation of oligodendrocyte precursor cells and myelin repair contributes to functional recovery following stroke [10]. These shared downstream mechanisms may provide additional biological support for applying neuroplasticity-based rehabilitation principles such as early mobilization, repetitive task-specific practice, and multidisciplinary rehabilitation to patients recovering from acute NMOSD relapse.

In our patient, intensive inpatient rehabilitation was initiated approximately three weeks after neurologic symptom onset in mid-April, after an initial hospitalization and SNF stay for aspiration pneumonia and clinically suspected prolonged NMOSD flare in late March. Consequently, the rehabilitation program occurred well within the proposed first 8-12-week window, during which neuroplastic reorganization is thought to be most responsive to task-specific rehabilitation. Functional improvement was observed across most Section GG domains by discharge, although the magnitude of improvement varied. The largest gains occurred in mobility domains (Table 2), particularly bed mobility (rolling, sit-to-lying, and lying-to-sitting), toilet and shower transfers, one-step curb negotiation, and activities that were initially not attempted because of medical or safety concerns, including car transfers and object retrieval. Self-care activities (Table 1), including toileting hygiene, bathing, lower extremity dressing, and footwear management, demonstrated smaller improvements and continued to require assistance at discharge.

​​​​​In contrast, more complex functional activities such as prolonged ambulation, uneven-surface walking, stair negotiation, and long-distance wheelchair propulsion were not attempted due to persistent safety concerns and residual neurologic deficits. Overall, the greatest functional gains occurred in foundational mobility and transfer activities, whereas more advanced mobility tasks remained appropriate targets for continued rehabilitation following discharge.

Objective neurologic findings supported functional improvements. Compared with admission, strength improved from 3-4/5 to 4/5 in the left lower extremity and from 4/5 to 4+/5 in the right upper and lower extremities. Ambulation was accompanied by less frequent left knee buckling and by improvements in both distance (from 28 to 35 feet) and assistance (from moderate to touch). Additionally, shoulder spasticity became transient and responded to medication adjustments by the time of discharge.

These neurologic improvements paralleled gains in mobility and self-care Section GG scores, suggesting that functional recovery reflected not only compensatory strategies but likely also improvement in underlying neurologic impairment. These improvements were clinically meaningful, as they translated into greater participation in activities of daily living and mobility-related tasks over the course of the admission. The improved mobility and self-care scores are important because they are associated with greater functional independence and may facilitate safer participation in home and community activities [14].

These improved functional outcomes align with findings from other stroke rehabilitation studies, which have shown improvements with early high-intensity therapy in patients with stroke [8,15]. Additional research shows that early mobilization and repetitive motor training enhance reactivation of the corticospinal tract and synaptic efficiency, leading to measurable improvements in motor control and ADL performance [5]. As previously stated, while NMOSD differs etiologically from stroke, both conditions result in sudden neurologic deficits and variable degrees of demyelination requiring neural reorganization to restore function. Thus, applying stroke rehabilitation principles to NMOSD recovery is both rational and promising.

Furthermore, this patient's clinical course highlights the potential benefits of inpatient rehabilitation facilities (IRFs) over skilled nursing facilities (SNFs) for neurologic recovery. Following her initial hospitalization and treatment with corticosteroids and trimethoprim-sulfamethoxazole, she was discharged to a SNF. However, she demonstrated minimal functional improvement during this period, as reflected by persistent left-predominant weakness, worsening gait instability characterized by left knee buckling, ambulation of only 28 feet with a front-wheel walker and moderate assistance, and decreased vision in the left eye. The lack of meaningful recovery at the SNF prompted transfer to an IRF, where she participated in a more intensive rehabilitation program.

IRFs provide structured, multidisciplinary care and typically require patients to participate in at least 3 hours of therapy per day, 5 days per week, similar to the patient in this case, who participated in approximately 3 hours of combined rehabilitation daily, consisting of approximately 120 minutes of PT, 60 minutes of OT, and daily rehabilitation nursing. This higher-intensity rehabilitation may facilitate neuroplasticity and functional recovery during a critical recovery window following neurologic injury [4,5,11].

In contrast, lower-intensity SNF environments have been associated with delayed functional gains and increased caregiver burden. A large cohort study by Hong et al. demonstrated similar findings in stroke populations, showing that among 99,000 patients, those discharged to IRFs achieved significantly greater improvements in mobility and self-care than those discharged to SNFs, even after adjusting for demographics and comorbidities [16]. These findings are derived from stroke populations and therefore cannot be assumed to apply directly to NMOSD. However, stroke rehabilitation principles may provide a reasonable conceptual framework for rehabilitation in NMOSD until disease-specific evidence becomes available because both conditions may produce abrupt neurologic deficits and substantial functional impairment. Although our patient's functional improvements are encouraging, they should be interpreted as observations from a single case rather than evidence of equivalent rehabilitation mechanisms or outcomes between the two conditions.

Another key finding from this case is the adaptability of the rehabilitation plan, despite the disease's volatility. Recent evidence supports continued modulation of rehabilitation management during NMOSD relapses rather than cessation of therapy. A prospective cohort study by Li et al. involving 73 patients with acute NMOSD and an Extended Disability Status Scale (EDSS) ≥ 4.5 demonstrated that early, structured rehabilitation, in addition to standard medical therapy, resulted in significantly greater improvements in EDSS, motor function, balance, and activities of daily living compared with controls. Among patients with moderately severe NMOSD, 90% achieved clinically meaningful improvement, compared with 27.8% in the control group (P < 0.05) [17]. Importantly, only 2 of 36 patients in the rehabilitation group experienced mild adverse events, characterized as slightly worsened fatigue or weakness, supporting the safety of early rehabilitation during acute disease. Additionally, a case report by Nishikawa et al. described a patient with severe acute NMOSD (EDSS 9.0) who began supervised PT as early as hospital day 4 and demonstrated marked functional recovery, including improvement in EDSS (9.0 to 6.0), motor strength, and functional independence measures, without clinical deterioration [18].

This evidence is consistent with our case, as our patient achieved functional progression during a suspected second flare and episodes of spasticity. The patient's rehabilitation course was supported by flexible therapy goals, the resumption of high-dose corticosteroids, and interdisciplinary coordination. Importantly, rehabilitation intensity was not maintained rigidly during periods of neurologic worsening. Therapy sessions were modified after close communication among the physiatrist, therapists, and nursing staff based on daily neurologic assessment, symptom progression, fatigue, and exercise tolerance. Some modifications included increased emphasis on stretching, shorter treatment durations as needed, and only one missed PT session during episodes of spasticity and fatigue. These adjustments were intended to balance the benefits of maintaining activity and preventing deconditioning with the need to minimize fatigue and symptom exacerbation. No sustained clinical deterioration attributable to participation in the modified rehabilitation was observed. As a result, the patient achieved better functional outcomes with only mild adverse events.

Additionally, this case also highlights the challenges of rehabilitation during periods of neurologic instability. Distinguishing disease progression, a new relapse, treatment-related fatigue, worsening spasticity, or overexertion during rehabilitation may be difficult because each can present with worsening weakness, fatigue, or reduced exercise tolerance. Also, as with other physiologic stressors encountered during recovery, increasing rehabilitation demands may coincide with symptom fluctuation or transient worsening, making ongoing clinical reassessment essential.

In our patient, rehabilitation was not discontinued during the clinically suspected second relapse; rather, therapy intensity was individualized with modifications based on the patient's daily neurologic status and tolerance. This adaptive approach allowed continued participation in rehabilitation while minimizing symptom exacerbation. It underscored the importance of close interdisciplinary communication and monitoring among the rehabilitation physician, therapists, and nursing staff to recognize evolving symptoms and promptly tailor rehabilitation.

However, a direct comparison between our patient and these two cohorts is limited because the patient's EDSS score was not formally assessed, making alignment with the study populations uncertain [17,18]. Although we did not measure EDSS scores, our case supports the notion that rehabilitation for NMOSD should not be paused during relapse but rather be modulated to maintain engagement, prevent deconditioning, and preserve neural pathways for recovery.

Conversely, relapses in NMOSD are associated with significant and often sustained declines in neurologic function and quality of life. Berthele et al., in a post hoc analysis of clinical trial data from the PREVENT study, demonstrated that even a single relapse in AQP4-positive NMOSD leads to clinically meaningful worsening in disability (EDSS, modified Rankin Scale) and health-related quality of life across multiple domains, with further deterioration observed with recurrent relapses [19]. These findings underscore the urgency of early and sustained rehabilitation following relapse, as each episode contributes to cumulative and potentially irreversible functional loss. Given the cumulative impact of relapse-related neurologic injury, optimizing functional recovery after each episode becomes critical. In this context, continued and adaptive rehabilitation may play a key role in mitigating functional decline, preventing secondary deconditioning, and preserving independence.

However, several limitations should be considered when interpreting these findings. As a single-patient case report, the observed outcomes may not be generalizable to the broader NMOSD population. The patient's disability was also not formally quantified using standardized measures such as the Expanded Disability Status Scale (EDSS) or a modified Rankin Scale, limiting direct comparison with existing NMOSD rehabilitation literature. Details regarding the patient's baseline maintenance immunotherapy, disease-modifying therapy, and long-term relapse frequency were unavailable in the transferred medical records, which limit characterization of her underlying disease course. Additionally, long-term follow-up data were unavailable, limiting assessment of the durability of functional gains after discharge, as subsequent outpatient care was documented outside the available medical record.

Furthermore, neither the initial episode nor the suspected second flare was confirmed using neuroimaging, cerebrospinal fluid analysis, or serologic testing. Consequently, the reproducibility of flare identification and the exclusion of alternative explanations for neurologic worsening are limited. Finally, the observational nature of this report precludes attributing the patient's functional improvement solely to the rehabilitation intervention, as recovery may have also been influenced by corticosteroid treatment, medical stabilization, or the natural course of the disease.

Despite these limitations, this case provides a clinically relevant example of how individualized, multidisciplinary rehabilitation can be integrated into the management of NMOSD during periods of neurologic recovery. The observed improvements in neurologic examination findings and Section GG functional scores support the feasibility of early, adaptive rehabilitation following clinically suspected NMOSD relapses. Although NMOSD and ischemic stroke differ in their underlying etiologies, shared downstream mechanisms of neural network disruption, neuroplasticity, and white matter repair provide a biologically plausible rationale for utilizing principles of early, task-specific rehabilitation across both conditions. Future studies are needed to define better optimal timing, intensity, and specific rehabilitation interventions that maximize neuroplastic recovery and sustained functional improvement in patients with NMOSD.

Collectively, this case report highlights the potential role of early, intensive inpatient rehabilitation in facilitating functional recovery following a clinically suspected NMOSD flare, with improvements in Section GG scores observed during the rehabilitation course. Given the rarity of NMOSD, adapting principles from stroke-based neurorehabilitation may provide a practical framework for supporting recovery and maximizing functional independence in patients with demyelinating disease.

## Conclusions

This case report highlights the potential role of early, intensive inpatient rehabilitation following a clinically suspected NMOSD flare. It also supports the feasibility of continuing individualized rehabilitation during periods of neurologic instability. The patient's substantial improvement in neurologic examination findings and Section GG functional scores over a two-week rehabilitation course illustrates meaningful functional recovery in early inpatient rehabilitation. Although causality cannot be established from a single case, these findings suggest that timely, multidisciplinary rehabilitation may provide a framework for functional recovery during the critical first 8-12-week neuroplastic recovery window following acute neurologic insult.

Although NMOSD and ischemic stroke differ in their underlying etiologies, shared downstream mechanisms of neuroplasticity and white matter repair provide a biologically plausible rationale for cautiously extrapolating selected rehabilitation principles of early, task-specific neurorehabilitation across both conditions. However, these findings should be interpreted with caution, as this report describes a single patient without long-term follow-up, standardized disability measures such as the Expanded Disability Status Scale (EDSS) or a modified Rankin Scale, or radiographic confirmation of the suspected flares. Further studies are needed to define better optimal timing, intensity, and long-term impact of rehabilitation interventions for patients with NMOSD.
